# Detection of lamivudine-resistant variants and mutations related to reduced antigenicity of HBsAg in individuals from the cities of Santos and São Paulo, Brazil

**DOI:** 10.1186/1743-422X-10-320

**Published:** 2013-10-28

**Authors:** Nathalia Mantovani, Maira Cicero, Luiz Claudio Santana, Carla Silveira, Eliane Pereira do Carmo, Paulo Roberto Ferreira Abrão, Ricardo Sobhie Diaz, Marcos Montani Caseiro, Shirley Vasconcelos Komninakis

**Affiliations:** 1 Retrovirology Laboratory, Infectious Diseases Division, Federal University of São Paulo, R. Pedro de Toledo, n. 781, 16° andar, São Paulo, SP Cep 04039-032, Brazil; 2 Department of Medicine, Biology Molecular Laboratory, Lusiada Foundation, Santos, Brazil; 3 Ambulatory of Center for Control of Immune Deficiencies, Federal University of São Paulo, São Paulo, Brazil

**Keywords:** HBV, Genotypes, Transmitted drug resistance, Lamivudine, Vaccine escape mutations, Polymerase gene, Surface gene, Surface antigen

## Abstract

**Background:**

Continuous long-term treatment is recommended to reduce the hepatitis B virus (HBV) viral load. However, as a consequence, resistance mutations can emerge and be transmitted to other individuals. The polymerase (POL) gene overlaps the surface (S) gene. Thus, during treatment, mutations in the POL gene may lead to changes in hepatitis B surface antigen (HBsAg). The purpose of this study was to evaluate the frequency of lamivudine and vaccine escape mutations in HBsAg-positive blood donors from the city of Santos and in untreated HBV mono-infected patients from the city of São Paulo, Brazil.

**Methods:**

HBV DNA was extracted from 80 serum samples, of which 61 were from volunteer blood donors and 19 were from untreated HBV patients. A fragment of the POL/S genes containing 593 base pairs was amplified using nested PCR. Thirty four were PCR-positive and sequencing was performed using an ABI Prism 3130 Genetic Analyzer. Alignments and mutation mapping were performed using BioEdit software.

**Results:**

HBV DNA from 21 blood donors and 13 untreated patient samples were characterized using nucleotide sequencing PCR products from the POL/S genes. We were able to detect one sample with the resistance mutation to lamivudine rtM204V + rtL180M (2.94%), which was found in a volunteer blood donor that has never used antiviral drugs. The other samples showed only compensatory mutations, such as rtL80F (5.88%), rtL80V (2.94%), rtL82V + rtV207L (2.94%), rtT128P (5.88%), rtT128N/S (2.94%) and rtS219A (5.88%). We found modifications in the S gene in 14 of the 34 samples (41.16%). The mutations detected were as follows: sM133L + sI195T (2.94%), sI195M (2.94%), sP120T (2.94%), sY100S/F (2.94%), sY100C (17.64%), sI/T126P + sQ129P (2.94%), sM198I + sF183C (2.94%) and sS210R (5.88%).

**Conclusions:**

Our results suggest the transmission of lamivudine-resistant forms. Thus, the evaluation of HBV-infected subjects for lamivudine resistance would improve treatment regime. Moreover, the mutations in the S gene may impair HBsAg antigenicity and contribute to HBsAg failure detection and vaccine escape.

## Background

Transmitted drug resistance (TDR) may impair or diminish a disease response to treatment by transmitting direct or cross-resistance among drugs. Resistance to lamivudine (LAM) increases the potential for cross-resistance to other nucleos(t)ide analogs (emtricitabine, telbivudine and clevudine and partial cross-resistance to entecavir), limiting treatment options [1]. There are few studies regarding the incidence of transmitted resistance to LAM. Moreover, the results differ among countries worldwide [2-5].

Resistance mutations caused by LAM can lead to changes in the amino acids in the surface antigen (HBsAg) because of the overlap between the polymerase (POL) and the surface (S) gene. These changes may compromise the patient’s response to the infection and to the vaccination [6,7].

Santos is the location of the most important seaport in Latin America. As such, it receives a large number of visitors from different countries and presents high levels of TDR to HIV, but there are no data about transmitted resistance to HBV [8-10]. The risk of contracting hepatitis B from blood transfusions in Brazil is 1 in 30,000 donations, and to our knowledge, no data on the distribution of HBV genotypes or changes in HBsAg are available for Santos. When combined with risk behaviors (drug use, men having sex with men and sex work), these factors may lead to an increased probability of the migration of LAM-resistant viruses and vaccine escape variants.

The city of São Paulo is considered the main financial center of Brazil and has a population from different ethnicities, which may influence the levels of resistance mutations and the distribution of HBV genotypes.

The aim of this study was to evaluate the frequency of lamivudine and vaccine escape mutations in HBsAg-positive blood donors from the city of Santos and in untreated HBV mono-infected patients from the city of São Paulo, Brazil.

## Results

### Genotypes

**Figure 1:**
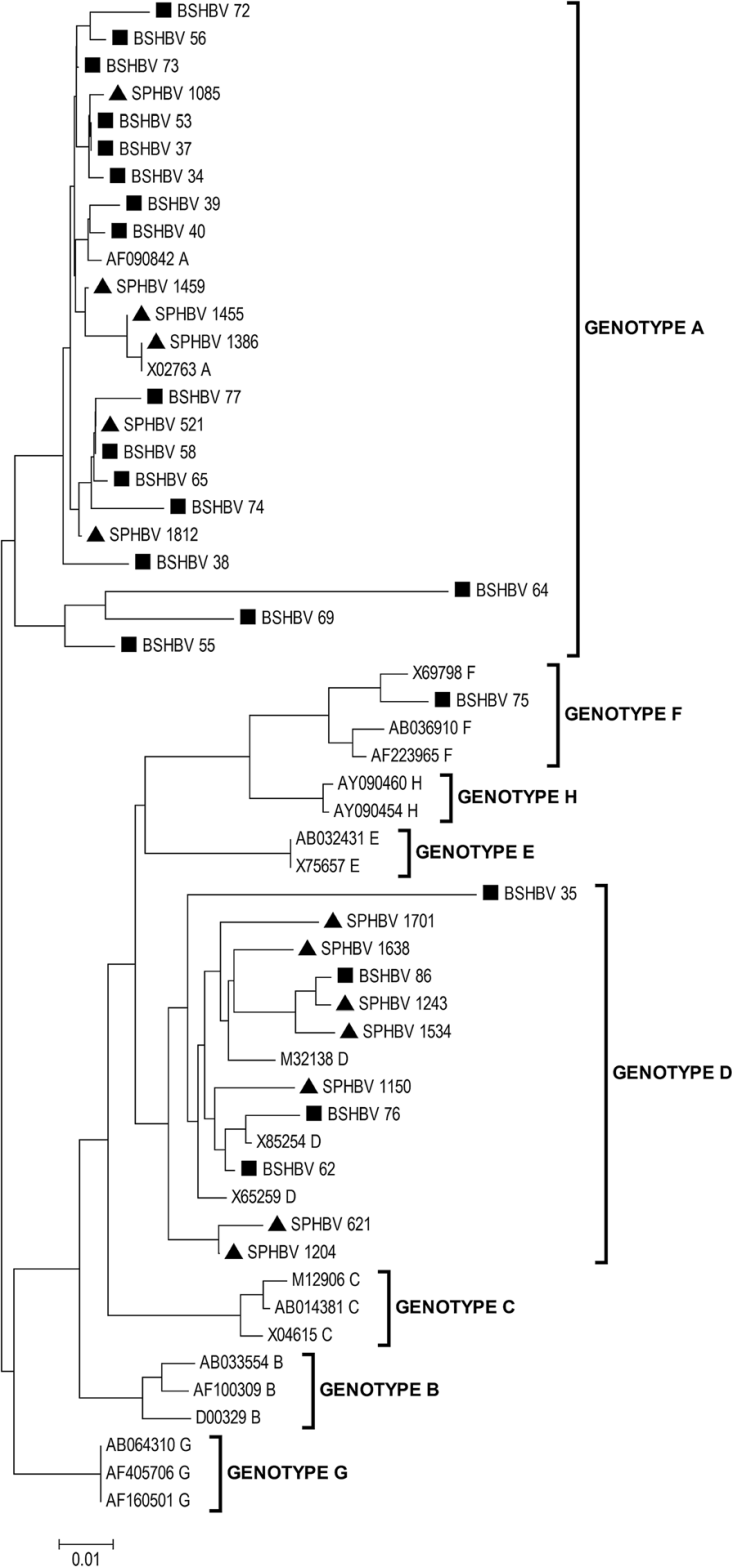
**Phylogenetic relationship generated from 34 HBV sequences.** A bootstrap analysis with 1,000 replications was performed to confirm the reliability of the tree. The approximate likelihood ratio test (aLRT) values ≥ 70% are indicated at the nodes. Nucleotide sequences were compared with reference sequences, which are indicated by accession number and alphabetical letter. Samples from the Blood Center of Santos are indicated with squares, and those from São Paulo are indicated with triangles.

Of the 34 samples analyzed, 64.7% had Genotype A, 32.35% had Genotype D, and 2.95% had Genotype F. These results are presented in the phylogenetic tree shown in Figure 1.

### Transmitted drug resistance mutations

Among the 13 subjects from São Paulo, we detected only the rtS219A + sS210R mutations. All the other modifications reported in the study were found in the 21 blood donor subjects from Santos. Modifications within the POL gene were observed in 9 out of 34 samples (26.47%); we found only one case with the rtM204V mutation, which caused complete resistance to LAM, along with the compensatory mutation rtL180M (2.94%). The other samples showed only the compensatory mutations rtL80F (5.88%), rtL80V (2.94%), rtL82V + rtV207L (2.94%), rtT128P (5.88%), rtT128N/S (2.94%) and rtS219A (5.88%).

### S Gene modifications

We found modifications in the S gene in 14 of the 34 samples (41.16%). The mutations detected were as follows: sM133L + sI195T (2.94%), sI195M (2.94%), sP120T (2.94%), sY100S/F (2.94%), sY100C (17.64%), sI/T126P + sQ129P (2.94%), sM198I + sF183C (2.94%) and sS210R (5.88%).

### Analyzing both genes together

When the genes were analyzed together, 9 of the 34 samples had mutations in both genes, and we found the following combinations: sM133L + sI195T with rtL80F + rtT128P, sI195M with rtL180M + rtM204V, sP120T with rtT128N, sY100F with rtT128S + rtL80V, sY100S with rtL80F, sY100C + sI/T126P + sQ129P with rtT128P, sM198I + sF183C with rtL82V + rtV207L and sS210R with rtS219A (in two samples), as shown in Table 1.

**Table 1:** **Mutations within the *pol *** **and *S *** **genes related to antiviral resistance, immune escape and diagnostic failure**

**ID**	**Subject**	**Genotype**	**Mutations in combination in both POL/S genes**	**Other mutations in POL and S gene**	**Impact of the S gene mutations**
35	BD	D	-	*rtL80F*, sI195T, sM133L*, rtT128P*	sI195T- Diagnostic failure and vaccine/HBIg therapy escape [11]
39	BD	A	**rtM204V +** *rtL180M*/sI195M	-	sI195M - Impair anti-HBs binding [7]
40	BD	A	rtT128N*/sP120T	-	sP120T – Vaccine/HBIg therapy escape [12]
55	BD	A	-	*rtL80F*, sY100S*	-
58	BD	A	-	sY100C*	-
62	BD	D	rtS219A*/sS210R*	-	-
64	BD	A	-	sI/T126P, sQ129P, rtT128P*, sY100C*	sI/T126P and sQ129P- Diagnostic failure [13]
65	BD	A	-	sY100C*	-
69	BD	A		*rtL80V***,** rtT128S*, sY100F*	-
74	BD	A	-	sY100C*	-
75	BD	F	*rtV207L*/sM198I	sF183C**, rtL82V*	sM198I - Reduced antigenicity of HBsAg [7]
					sF183C**- Reduced anti-HBs binding [9]
77	BD	A	-	sY100C*	-
521	UP	A	-	sY100C*	-
1150	UP	D	rtS219A*/sS210R*	-	-

Mutation in bold: Primary mutation related to Lamivudine resistance; Mutations in italic: Compensatory mutations; Underlined Mutations: Have clinical significance; *No in vitro assays; ** Natural polymorphism in the genotype F; ID: Sample identification; BD: Blood Donor; UP: Untreated Patient; HBsAg- Hepatitis B Surface Antigen; POL: Polymerase gene; S: Surface gene; HBIg- Immunoglobulin against Hepatitis B Virus.

## Discussion

The frequencies of the genotypes found in our study are in agreement with other studies showing Genotype A as the most frequent, followed by D and F, in southeastern Brazil [8]. The Genotype F is endemic in Northern Brazil [14], nevertheless we indentified this genotype in one sample from Santos.

The genotype distribution found in the study reflects the historical context of the Brazilian population, which is composed of Amerindians and immigrants from Africa and Europe, although the migration of people from north and northeast Brazil to southeast also occurs [8]. Although we were not able to correlate the genotypes with the mutations detected, probably because of the small number samples analyzed, we found the mutation sF183C in an individual infected with genotype F, which corroborates with other studies that found that this mutation is a natural polymorphism for genotype F and H [9].

The primary mutation rtM204V reduces susceptibility to LAM and decreases viral fitness, which is restored by compensatory mutations [1]. We found the transmitted mutation rtM204V (2.94%) with the compensatory mutation rtL180M in a blood donor sample. This individual has never received antiviral treatment; however, we are not able to inform if this blood donor was vaccinated against HBV, since this issue is not addressed in the specific questionnaire used during the donor screening.

Variants harboring both primary and secondary mutations may not substantially reduce their fitness because the secondary mutations are capable to rescue the fitness loss caused by the primary mutations [10,14,15]. Then, it is conceivable that we were able to detect the rtM204V variant because it was associated with the compensatory mutation at position rt180 (rtL180M), which may recover its fitness and stability *in vivo*. In fact, a chimpanzee study performed by Kamili et al., reported a successful infection by HBV mutants harboring the mutations rtM204V + rtL180M, which corroborates our finding that these variants can be infectious and stable [16].

To our knowledge, no previous studies have evaluated TDR in untreated HBV mono-infected individuals in Brazil. However, among chronically infected and treated Brazilian individuals, the rate of LAM resistance (rtM204V + rtL180M) ranges from 16% to 41% [17,18]. Regarding HBV/HIV co-infected and treated individuals, the mutation rate ranges from 21.42% to 45.45% [19-21].

Mutations in the S gene may lead to diagnostic failure and vaccine escape because the specific antibodies produced against HBsAg may not recognize the antigens produced by the mutated S gene. These mutations were detected in almost half of the individuals enrolled in our study, although the role of some of these mutations in clinical practice is not well understood, as shown in Table 1. Lai et al. (2012) also found a high level of individuals infected with HBsAg mutants, from which five out of eight individuals vaccinated against HBV and positive for HBV DNA were infected with variants harboring S gene mutations. In addition, they found an increase in the detection of HBV serological markers in adults compared with children and adolescents, suggesting that HBV infection in adults may need new strategies for prevention [22]. sY100C has been reported in cases of occult hepatitis, however Mello et al. (2011) found that this mutation did not have any impact for HBsAg expression and affinity by commercial serologic assays, which is in agreement with our results that found this mutation in five HBsAg-positive blood donors [23]. When the POL and S genes were analyzed together, we found mutations in both genes that can be explained by their overlapping reading frames in the HBV genome. Thus, changes at one position may affect the structure of more than one viral protein. Although we used conventional sequencing technology, among blood donors and untreated patients, we observed one mutation that led to loss of susceptibility to LAM as well as some compensatory mutations capable of restoring viral fitness. Nevertheless, it is worth noting that viral particles harboring S mutations have a decrease in viral protein expression and are less infectious when compared with wild type strains, as observed in *in vitro* assays [24]. Because resistance mutations can rapidly decrease following the withdrawal of therapy and the absence of drug selection pressure, it would be interesting to use more accurate methods, such as next-generation sequencing, to detect resistance mutations [25].

## Conclusions

We observed transmitted resistance mutations in a small population of HBsAg-positive blood donors and untreated HBV mono-infected patients. Although we found circulation of POL and S gene mutations, the persistence of these variants in the absence of therapy is not completely elucidated. Further studies are necessary to clarify the impact of these mutations for real use in clinics and prevention.

## Methods

Eighty samples were collected, of which 61 were from blood donors and 19 were from HBV untreated patients, between 2008 and 2009 at Santos Blood Center and between 2007 and 2011 in São Paulo city, respectively. All of the patients were mono-infected with HBV (HBsAg positive) and had not received prior treatment with nucleos(t)ide analogs. The study was approved by the Ethics Committees and the Institutional Review Board of the Federal University of Sao Paulo (#0485/09) and Lusiada Foundation of Santos (#059/2010). Because all blood donor data were confidential, we did not have access to demographic or risk behavior data for these subjects. Regarding the HBV mono-infected patients, 54% (7) were female, 46% (6) were male, and the average age was 41 years.

HBV DNA was extracted from serum using a QIAamp Blood Mini Kit (Qiagen, Valencia, CA, USA). A 593-bp DNA fragment encompassing the HBV POL and S genes was amplified using nested PCR [26]. Positive and negative controls were included at all stages of PCR. Sequencing was performed using an ABI Prism 3130 Genetic Analyzer (Applied Biosystems Inc., Foster City, CA). Alignments were performed using BioEdit Sequence Alignment v. 7.1.5 software [27] and the reference sequence NC003977.1. Phylogenetic relationships among the individual sequence types were determined using the neighbor-joining algorithm of MEGA v.4 [28]. The frequencies of resistance mutations were calculated using Fisher’s exact test (Minitab v. 16) [29].

## Abbreviations

DNA: Deoxyribonucleic acid; HBsAg: Hepatitis B surface antigen; HBV: Hepatitis B virus; HIV: Human immunodeficiency virus; LAM: Lamivudine; PCR: Polymerase chain reaction; POL: Polymerase; S: Surface; TDR: Transmitted drug resistance.

## Competing interests

The author’s declare that they have no competing interests.

## Authors’ contributions

N. M. – AB, JY, MT, ES, FG. M. C. – AB, JY, MT, ES, FG. L.C. S. – AB, JY, MT, ES, FG. C. S. – JY. E. P. d. C. – MT. P. R. A. F. – MT. S. C. V. K. – FG. All authors read and approved the final manuscript.
